# Detecting Retinal Nerve Fibre Layer Segmentation Errors on Spectral Domain-Optical Coherence Tomography with a Deep Learning Algorithm

**DOI:** 10.1038/s41598-019-46294-6

**Published:** 2019-07-08

**Authors:** Alessandro A. Jammal, Atalie C. Thompson, Nara G. Ogata, Eduardo B. Mariottoni, Carla N. Urata, Vital P. Costa, Felipe A. Medeiros

**Affiliations:** 1 Vision, Imaging and Performance Laboratory (VIP), Duke Eye Center and Department of Ophthalmology, Duke University, Durham, NC USA; 2 Department of Ophthalmology, University of Campinas, Campinas, Brazil

**Keywords:** Medical imaging, Optic nerve diseases

## Abstract

In this study we developed a deep learning (DL) algorithm that detects errors in retinal never fibre layer (RNFL) segmentation on spectral-domain optical coherence tomography (SDOCT) B-scans using human grades as the reference standard. A dataset of 25,250 SDOCT B-scans reviewed for segmentation errors by human graders was randomly divided into validation plus training (50%) and test (50%) sets. The performance of the DL algorithm was evaluated in the test sample by outputting a probability of having a segmentation error for each B-scan. The ability of the algorithm to detect segmentation errors was evaluated with the area under the receiver operating characteristic (ROC) curve. Mean DL probabilities of segmentation error in the test sample were 0.90 ± 0.17 vs. 0.12 ± 0.22 (P < 0.001) for scans with and without segmentation errors, respectively. The DL algorithm had an area under the ROC curve of 0.979 (95% CI: 0.974 to 0.984) and an overall accuracy of 92.4%. For the B-scans with severe segmentation errors in the test sample, the DL algorithm was 98.9% sensitive. This algorithm can help clinicians and researchers review images for artifacts in SDOCT tests in a timely manner and avoid inaccurate diagnostic interpretations.

## Introduction

Spectral-domain optical coherence tomography (SDOCT) is the most commonly used diagnostic tool for detection of structural changes from glaucoma and other non-glaucomatous optic neuropathies^1,2^. SDOCT acquires measurements of the thickness of the peripapillary retinal nerve fibre layer (RNFL) at a micrometre scale that have been successfully used for both diagnosis and detection of disease progression^3–7^.

Assessment of the RNFL thickness with SDOCT currently requires accurate delineation of the anterior and posterior boundaries of the RNFL. However, despite advances in SDOCT hardware and software, errors in segmentation of the RNFL are still relatively common. According to the literature, anywhere from 19.9% to 46.3% of SDOCT scans of the RNFL contain artifacts or segmentation errors^8–12^. Segmentation errors may lead to spurious measurements of the RNFL thickness, potentially increasing false-positives and false-negatives as well as leading to increased test-retest variability and diminished ability to detect disease progression over time.

Recent progress in artificial intelligence and machine learning has made it possible to develop sophisticated algorithms for image assessment that can generally replicate skilled human evaluations or sometimes even outperform them. Examples include deep learning (DL) algorithms trained to detect signs of diabetic retinopathy and glaucoma on fundus photographs as well algorithms to detect retinal lesions on OCT^13–17^. It is conceivable that such algorithms could similarly be trained to identify errors in automated segmentation of SDOCT scans. Such an algorithm could help clinicians and researchers rapidly identify whether these errors are present and whether the measurements could be considered reliable or not. In the present study, we propose and validate a novel DL algorithm to detect segmentation errors on SDOCT scans for RNFL assessment.

## Results

The dataset included 25,250 SDOCT B-scans from 1,363 eyes of 709 subjects. The mean age of subjects was 61.7 ± 15.4 years, and 375 (56.2%) were female. Approximately 65.3% were white race and 34.7% self-identified as black. Also, 407 (29.9%) eyes were classified as glaucomatous, 575 (42.1%) as suspect, and 381 (28.0%) as healthy. From the 25,250 SDOCT scans, 2,597 (10.3%) had an RNFL segmentation error according to human graders. 422 eyes of 316 patients had at least 1 scan with segmentation error. From the 2,597 scans with segmentation errors, 1,895 (73.0%) were classified as mild and 702 (27.0%) as severe errors by human graders. The dataset was split into training plus validation (50%) and test (50%) samples (Table 1).

**Table 1:** Demographic and clinical characteristics of the eyes and subjects included in the training and test samples by randomization at the patient level.

	Normal	Suspect	Glaucoma	Overall
**Training** + **Validation sample**				
Number of eyes	178	291	213	682
Number of patients	94	160	100	354
Number of images	1,900	5,897	5,334	13,131
Number of images with errors	135	394	834	1,363
Age (years)	48.9 ± 16.4	64.9 ± 11.6	70.7 ± 11.1	62.3 ± 15.4
Female gender (%)	54.4	58.0	49.4	54.7
Race (%)				
Caucasian	71.3	68.1	56.0	65.5
African-American	28.7	31.9	44.0	34.5
SAP MD (dB)	0.04 ± 1.25	−0.45 ± 1.81	−6.16 ± 6.2	−2.70 ± 5.06
SDOCT Average RNFL Thickness (μm)	98.3 ± 9.8	86.4 ± 13.4	71.6 ± 16.9	82.1 ± 17.3
**Test sample**				
Number of eyes	203	284	194	681
Number of patients	104	153	98	355
Number of images	2,787	4,727	4,605	12,119
Number of images with errors	155	225	854	1,234
Age (years)	50.0 ± 16.3	63.4 ± 12.2	69.3 ± 12.1	61.1 ± 15.5
Female gender (%)	57.3	61.6	51.7	57.6
Race (%)				
Caucasian	49.0	75.8	63.3	64.5
African-American	51.0	24.2	36.7	35.5
SAP MD (dB)	−0.06 ± 1.19	−0.25 ± 1.96	−6.48 ± 6.71	−2.58 ± 5.32
SDOCT Average RNFL Thickness (μm)	97.9 ± 10.6	87.3 ± 13.9	71.2 ± 17.5	83.6 ± 18.1

SAP = Standard Automated Perimetry; MD = Mean Deviation; SDOCT = Spectral-Domain Optical Coherence Tomography; RNFL = Retinal Nerve Fibre Layer.

The test sample consisted of 12,119 scans, with 1,234 (10.2%) classified as having at least one RNFL segmentation error (Table 2). Scans with segmentation error(s) had similar mean quality scores compared to those that did not have a segmentation error (27.2 ± 4.2 vs. 27.0 ± 4.7; P = 0.538; GEE). The mean global RNFL thickness was significantly lower in scans with segmentation error(s) compared to those without error(s) (71.2 ± 25.5 μm vs. 85.0 ± 16.5 μm, P < 0.001; GEE). Of note, segmentation errors were also significantly more frequent in eyes with glaucoma (P = 0.048; GEE), those with lower standard automated perimetry (SAP) mean deviation (MD; P < 0.001 GEE), thinner RNFL thickness (P < 0.001; GEE) or older age (P < 0.001; GEE).

**Table 2:** Characteristics of the eyes and subjects in the test sample according to the presence of segmentation errors, as classified by human graders.

	B-scans without segmentation errors (n = 10,885)	B-scans with segmentation errors (n = 1,234)	P-value
Number of eyes	629	52	—
Number of patients	327	28	—
Age at time of scan (years)	64.1 ± 13.9	69.5 ± 12.4	**<0.001** ^**a**^
Diagnosis (%)			**0.048** ^**a**^
Normal	31.8	5.8	
Suspect	42.8	28.8	
Glaucoma	25.4	65.4	
Female gender (%)	58.8	42.9	0.113^b^
Race (%)			0.682^b^
Caucasian	65.1	60.7	
African-American	34.9	39.3	
SAP MD (dB)	−2.08 ± 4.67	−6.99 ± 7.98	**<0.001** ^**a**^
SDOCT Quality score	27.0 ± 4.7	27.2 ± 4.2	0.538^a^
SDOCT Average RNFL Thickness (μm)	85.0 ± 16.5	71.2 ± 25.5	**<0.001** ^**a**^
Mean DL probability of a segmentation error	0.12 ± 0.22	0.90 ± 0.17	**<0.001** ^**a**^

SAP = Standard Automated Perimetry; MD = Mean Deviation; SDOCT = Spectral-Domain Optical Coherence Tomography; RNFL = Retinal Nerve Fibre Layer.

Values given as mean ± standard deviation, unless otherwise noted.

^a^Generalized estimating equation; ^b^Fisher’s exact test.

The DL algorithm was trained to output the probability of a segmentation error. Mean DL probabilities of a segmentation error in the test sample were 0.90 ± 0.17 vs 0.12 ± 0.22 (P < 0.001) for scans with and without segmentation error(s) as defined by human graders, respectively. The DL algorithm had an area under the ROC curve of 0.979 (95% CI: 0.974 to 0.984) with an overall accuracy of 92.4%. For a probability cut-point of 0.5, the DL algorithm was 95.0% sensitive and correctly identified 1,172 of the 1,234 scans that had any segmentation error(s) in the test sample. Conversely, the DL algorithm correctly classified 10,025 of 10,885 scans that did not have any segmentation error, achieving an overall specificity of 92.1%. Of the 360 scans with severe segmentation errors in the test sample, the DL algorithm was 98.9% sensitive. Also, the DL algorithm was 93.4% (N = 816/874) sensitive for mild segmentation errors. The performance of the DL algorithm in detecting segmentation errors was not significantly different among normal and suspect eyes and glaucoma eyes (ß = −0.141; 95% CI: −0.588 to 0.443). There was also no significant impact of signal strength on the ROC curve area (ß = −0.019; 95% CI: −0.063 to 0.021).

**Figure 1:**
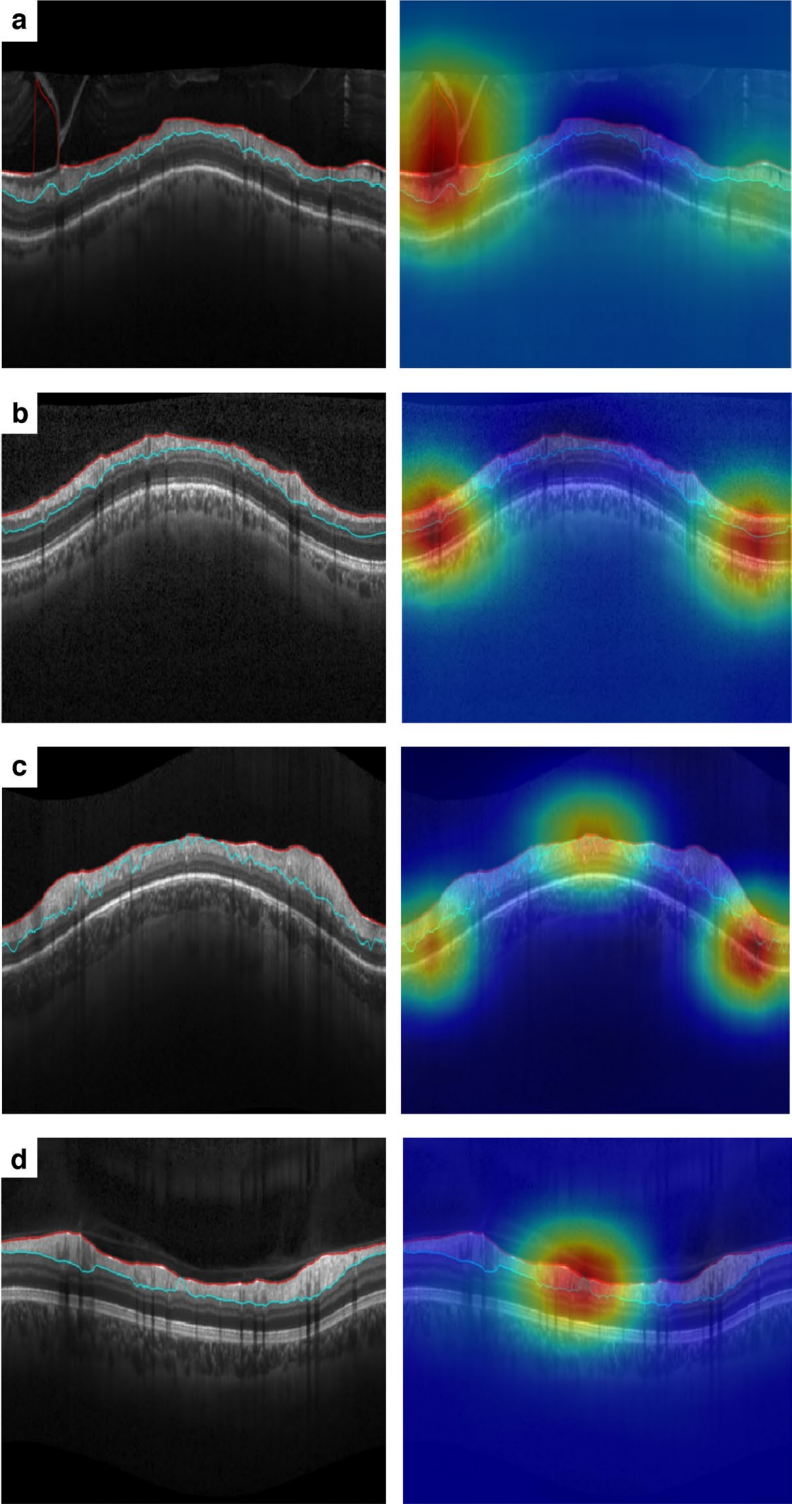
Spectral-domain optical coherence tomography (SDOCT) B-scans with segmentation errors correctly detected by both human graders and the deep learning algorithm. Class activation maps (heatmaps) on the right show the regions of the B-scans that had greatest weight in the deep learning algorithm classification. Probabilities of segmentation error given by the deep learning algorithm were all above 97.5% for these scans. (**a**) Segmentation error in the internal limiting membrane. (**b**) Segmentation error in the inner plexiform layer. (**c**) Multiple segmentation errors. (**d**) Small segmentation error.

Figure 1 provides several examples of B-scans with RNFL segmentation errors identified by human graders that were also correctly identified by the DL algorithm. The heatmaps illustrate the activation areas that were most important in the algorithm’s classifications. The DL algorithm detected errors of segmentation involving both the delineation of the internal limiting membrane (Fig. 1a) as well as the posterior boundary of the RNFL (Fig. 1b). Also, multiple errors could be detected in the same scan (Fig. 1c), as well as very small segmentation errors (Fig. 1d).

**Figure 2:**
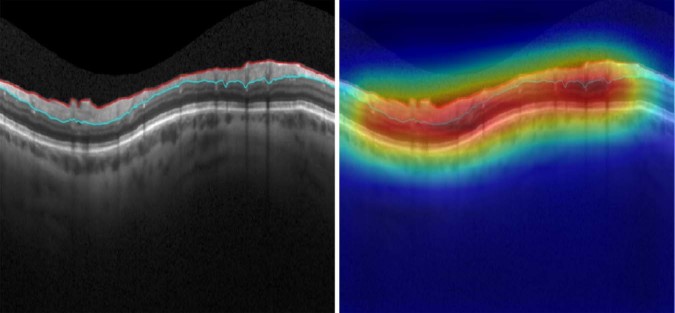
Spectral-domain optical coherence tomography (SDOCT) B-scan with no segmentation error, as labelled by human graders and the deep learning algorithm. Class activation map (heatmap) involves the whole B-scan, rather than being concentrated on a particular area. The probability of segmentation error given by the deep learning algorithm was below 1%.

Figure 2 shows an example of a scan that had no segmentation error according to human graders and the DL algorithm classification. One can see that the activation area (heatmap) involves the whole SDOCT scan in the figure, rather than concentrating on a particular region. This is expected as all areas of the RNFL segmentation were equally important in reaching the decision that no error was present.

**Figure 3:**
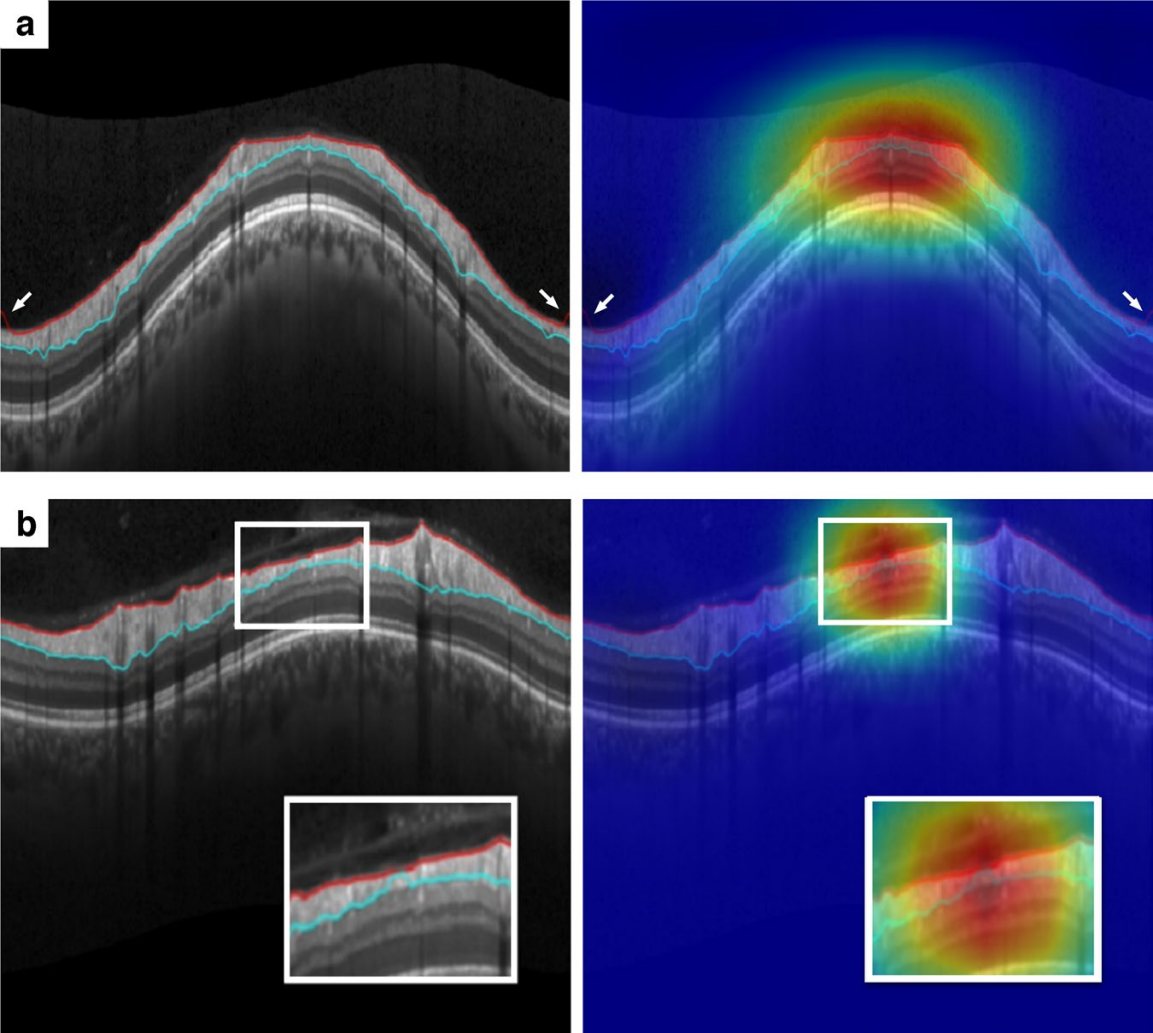
Spectral-domain optical coherence tomography (SDOCT) B-scans with disagreement about the presence of segmentation errors between human graders and the deep learning algorithm. (**a**) Small segmentation error in the internal limiting membrane occurring at the borders of the image (arrows) missed by the deep learning algorithm. The probability of segmentation error given by the deep learning algorithm was 15%. (**b**) Small segmentation error (detail) missed by the human labelling but identified by the deep learning algorithm. The probability of segmentation error given by the deep learning algorithm was 91%.

Figure 3 illustrates examples of misclassifications of the DL algorithm compared to the human graders. In Fig. 3a, the DL algorithm missed small segmentation errors from the ILM occurring at the borders of the scan (arrows). Figure 3b shows a scan that was classified as without error by human graders but received a probability of 0.91 of error from the DL algorithm. Close observation of the scan reveals that there is a very small segmentation error that was missed by the human graders.

## Discussion

In the present study, we introduce a novel DL algorithm that assesses SDOCT B-scans for segmentation errors of the retinal nerve fibre layer. The supervised algorithm was trained to output the probability of a segmentation artifact as well as highlight the location of these errors with a heatmap. Overall the algorithm was 92.4% accurate in images that had been evaluated by human graders. To the best of our knowledge, we are the first to train an algorithm capable of detecting RNFL segmentation errors on SDOCT B-scans. This algorithm may help clinicians and researchers to identify segmentation errors with high accuracy, which may impact diagnosis and detection of progression in glaucoma.

SDOCT has become the *prima facie* standard for objective quantification of structural damage in glaucoma due to its high reproducibility and accuracy^3–5^. However, artifacts during automated segmentation of the RNFL on SDOCT are not uncommon. Several studies have reported high rates of errors on segmentation of the RNFL across different SDOCT instruments^8–11,18,19^. Since most of the cohort studies in the literature that reported on SDOCT for glaucoma diagnosis excluded images with artifacts, the rate of segmentation errors could be substantially higher in routine clinical practice^8^.

The reasons for segmentation artifacts on SDOCT are multifactorial. Automated segmentation can be impaired by pathologic conditions that alter the normal shape of the retinal layers. Asrani and colleagues^8^ identified three common sources of RNFL imaging artifacts: posterior vitreous detachments, high myopia, and epiretinal membranes, with the latter being the most common culprit. Since SDOCT acts as a map of the reflectivity of the sample, any prominent abnormal hyperreflective band in the vitreoretinal interface may be misidentified as the internal limiting membrane retinal boundary, resulting in an overestimation of the retinal thickness^19^.

Additionally, SDOCT has inherent characteristics that can impair correct image segmentation^20–23^. First, SDOCT tomograms suffer from intrinsic speckle noise, which degrades the image quality and complicates image analysis^24^. Second, with increasing imaging depth there is increasing absorption and scattering of light in the retinal tissue. Third, optical shadows of the retinal blood vessels often weaken the gradation of intensities between retinal layers. Also, operator-related artifacts and sub-optimal scan quality reduce the accuracy of automated segmentation. Mansberger *et al*.^9^ observed that manual refinement changed 298 of 3,486 (8.5%) scans to a different global glaucoma classification, while 146 of 617 (23.7%) borderline classifications became normal. However, the authors excluded scans from their analysis that demonstrated distortion of the RNFL by vitreous traction or epiretinal membranes, which are common sources of segmentation errors. Thus, the probability that segmentation errors would affect diagnostic category is likely even greater in a clinical setting. We also noted that segmentation errors were more likely to occur in eyes with thinner global RNFL thickness, which has been previously observed by several other groups^8,10,12,18,19,25^. This finding highlights the importance of correctly identifying segmentation errors in patients with glaucoma and other optic neuropathies, where errors can adversely impact clinical decision-making.

Despite widespread adoption of SDOCT in clinical practice, many clinicians have not been trained to identify artifacts on SDOCT. Moreover, as many as 12.7% of artifacts may not be obvious on the final printout used by clinicians^8^. Consequently, they also may not be in the practice of manually correcting segmentation errors as they occur^8,26^. Development of methods to detect such errors would greatly benefit clinicians, especially as patient volume continues to rise. Similarly, in research settings, the manual labelling of segmentation errors on SDOCT by masked graders and reading centers is both time-consuming and expensive. Machine-aided assessment of SDOCT scans could facilitate the timely and accurate identification of such errors and potentially help to mitigate related misdiagnoses.

We applied a supervised DL algorithm with human gradings of SDOCT image quality serving as the reference standard. In our test sample, segmentation errors were identified by human graders in 10.2% of the images, and the DL classifier agreed with this classification in 95.0% of these scans at a probability cut-point of 0.5. Importantly, the algorithm correctly identified 98.9% of cases of segmentation errors that were deemed severe by human graders. This is important since such severe errors are most likely to prevent accurate estimation of RNFL thickness. The activation heatmaps demonstrated that the most important locations in the B-scans for the DL algorithm classifications corresponded precisely to the areas of error in segmentation (Fig. 1).

The establishment of a reference “ground truth” for training DL algorithms remains a challenge due to the fact that human gradings themselves may be subject to false-negatives and false-positives. In our study, we attempted to mitigate this by the use of a standardized approach to image quality grading. However, artifacts could have been missed during the original labelling of the scans by human graders, resulting in an artificially lower specificity of the algorithm. In fact, Fig. 3b shows an example where the deep learning algorithm identified a subtle artifact that had been missed by the human graders. Such a finding raises the possibility that DL algorithms may be able to outperform human graders, even when trained with an imperfect reference standard generated by human labelling of images. External assessment of the validity of our test results in research and clinical settings will be an important next step.

This study has limitations. The algorithm was only trained to indicate segmentation errors from RNFL circle B-scans. Future work should also include other types of scans available from SDOCT, such as those assessing the ONH and macular regions. Another consideration is that the algorithm was developed on images acquired from one specific model of SDOCT. Although different manufacturers have developed unique proprietary software for RNFL segmentation, there is some standardization in the reporting (i.e., RNFL thickness profile and delineation of its boundaries in B-scans), which may allow the generalization of this algorithm for other devices. Future studies should validate the algorithm in different instruments. Finally, while our results are promising, it is likely that the algorithm’s performance may be further refined by training on even larger datasets. Of note, in one case the algorithm appeared to have difficulty in identifying the segmentation errors along the borders of the image (Fig. 3a), and it is possible that this may be due to the low number of such cases in the training set.

In conclusion, we developed a DL algorithm that can accurately and quickly identify errors in the segmentation of the RNFL on SDOCT B-scans. This algorithm can help clinicians and other practitioners avoid inaccurate interpretations that result from automated segmentation errors. Application of such an algorithm in clinical practice and research settings may augment one’s ability to distinguish true RNFL thinning in glaucoma from artifact, and thus improve decision-making for disease management.

## Methods

This was a cross-sectional study with data drawn from the Duke Glaucoma Repository, a database of electronic medical and research records developed by the Vision, Imaging and Performance (VIP) Laboratory at the Duke Eye Center. The Institutional Review Board approved this study, with a waiver of informed consent due to the retrospective nature of this work. All methods adhered to the tenets of the Declaration of Helsinki for research involving human subjects and were conducted in accordance with regulations of the Health Insurance Portability and Accountability Act (HIPAA).

The database contained information on comprehensive ophthalmologic examinations during follow-up, diagnoses, medical history, visual acuity, slit-lamp biomicroscopy, intraocular pressure measurements, results of gonioscopy, and dilated slit-lamp funduscopic examinations. In addition, the repository contained Spectralis SDOCT (Software version 5.4.7.0, Heidelberg Engineering, GmbH, Dossenheim, Germany) images and standard automated perimetry (SAP) acquired with the 24-2 Swedish interactive threshold algorithm (Humphrey Field Analyzer II, Carl Zeiss Meditec, Inc., Dublin, CA).

Diagnosis of glaucoma was defined based on the presence of repeatable (at least two consecutive) abnormal SAP results with corresponding optic nerve damage. Abnormal SAP results were defined as a pattern standard deviation with P < 0.05, glaucoma hemifield test results outside normal limits, or both. Normal subjects had a normal optic disc appearance on slit-lamp fundus examination in both eyes, no history of intraocular pressure >21 mmHg, and normal SAP results. Glaucoma suspects had a history of elevated intraocular pressure, suspicious appearance of the optic disc on slit-lamp fundus examination, or other risk factors for the disease with normal SAP. Patients were excluded if they had a history of other ocular or systemic diseases that could affect the optic nerve or the visual field.

### Spectral-domain optical coherence tomography

Images of the peripapillary RNFL were acquired using the Spectralis SDOCT. The device has been previously described in detail^27^. The device employs a dual-beam SDOCT and a confocal laser-scanning ophthalmoscope with a super luminescent diode light (centre wavelength of 870 nm) as well as an infrared scan to provide simultaneous images of ocular microstructures. The peripapillary 12-degree circular optic nerve head (ONH) scan with 100 averaged consecutive circular B-scans (diameter of 3.45 mm, 1536 A-scans) was used for this study. The centre of rotation for the B-scans was the centre of the ONH as it appeared within the infrared fundus image acquired at the time of SDOCT B-scan relative to the angle between the fovea and the centre of Bruch’s membrane opening. Corneal curvature and axial length measurements were entered into the instrument’s software to ensure accurate scaling of all measurements. In addition, the device’s eye-tracking capability was used during image acquisition to adjust for eye movements.

SDOCT images were manually reviewed to assess quality, scan centration and segmentation errors following a standardized protocol. The anterior and posterior RNFL boundaries in the circle scan corresponded to the inner limiting membrane and the inner plexiform layer, respectively, as automatically delineated by the Spectralis SDOCT software. These boundaries are found by a threshold procedure, in which differences in reflectance between outer and inner retinal structures are interpreted as different layers^28^. Scans were classified as having an error in the RNFL segmentation versus having “no segmentation error”. Those that exhibited an RNFL segmentation error were further subjectively classified as “mild” or “severe” according to whether they would produce mild or severe impact on the RNFL thickness measurements. No manual correction of segmentation was made to the B-scans. All images had a quality score greater than 15 and images that were inverted or clipped were excluded.

### Image processing and development of the deep learning algorithm

A DL algorithm was trained to predict SDOCT segmentation errors from assessment of SDOCT B-scans. The target value was the probability of the presence of an RNFL segmentation error in a B-scan. Therefore, for training the neural network, all B-scans received a single binary label indicating the presence or not of a segmentation error. The whole dataset was then randomly split at the patient level into a training plus validation set (50% of the sample). A test sample (50%) was used to test the DL algorithm and yield probability scores of a segmentation error. In order to prevent leakage and biased estimates of test performance, no data for a given patient was present in both the training plus validation and the test samples.

SDOCT B-scans were initially pre-processed to derive data for the DL algorithm. The images were downsampled to 496 × 496 pixels and pixel values were scaled to range from 0 to 1. Data augmentation, which is a method of image transformation, was performed to increase the heterogeneity of the photographs, reduce the possibility of overfitting, and allow the algorithm to learn the most relevant features. Data augmentation included the following: random lighting which included changes in image balance and contrast of up to 5%, random rotations of up to 10 degrees in the image, and random horizontal flips of the image.

In the present work, we used a Residual deep neural Network (ResNet34) architecture that had been previously trained on the ImageNet^29^ dataset. The ResNet allows relatively rapid training of very deep convolutional neural networks by identifying shortcut connections that skip one or more layers and decrease the vanishing gradient issue when training deep networks^30^. As the recognition task of the present work largely differs from that of ImageNet, further training was also performed by first unfreezing the final 2 layers. Next, all layers were unfrozen, and training was performed using differential learning rates, in which different learning rates are used for different parts of the network such that a lower rate is used for the earlier layers and is gradually increased in the later layers. The network was trained with minibatch gradient descent of size 64 and Adam optimizer^31,32^. The optimal learning rate was achieved using the cyclical learning method with stochastic gradient descents with restarts^33^.

Gradient-weighted class activation maps (heatmaps)^34,35^ were also built over the input images to indicate the importance of each location of the image to the class under consideration. This technique allows one to visualize the parts of the image that are most important to explain the deep neural network classification.

### Statistical analyses

We evaluated the performance of the DL algorithm in the test sample by comparing the output predictions of the model with the human labelling of the SDOCT B-scans for the presence or absence of segmentation errors. Receiver operating characteristic (ROC) curves were used to assess the ability of the deep learning algorithm to identify segmentation errors that had been previously identified by human graders. The ROC curve is a plot of sensitivity versus 1 – specificity. Diagnostic accuracy of the algorithm was summarized by the area under the receiver operating characteristic curve (AUC) with 95% confidence intervals. For AUC, a value of 1.0 represents perfect discrimination, whereas a value of 0.5 represents chance discrimination. ROC regression was used to analyse the effects of covariates, such as signal strength and clinical diagnosis (i.e., normal and glaucoma suspect eyes vs. glaucoma eyes) on the performance of the DL algorithm. A ß-coefficient greater than zero for a specific covariate implies a positive discrimination between those with the characteristic or with increasing values of this covariate^36^. Generalized estimating equations (GEE) were used to account for the fact that multiple SDOCT images were obtained per patient. A bootstrap resampling procedure was used to generate 95% confidence intervals and P-values in which the standard errors were adjusted to account for the use of multiple images of both eyes in the same participant. This approach has been previously described as a way to adjust for the presence of multiple correlated measurements within the same unit^36,37^.

## Data Availability

The datasets generated during and/or analyzed during the current study are available from the corresponding author on reasonable request.
